# Mechanism of muscle atrophy in a normal-weight rat model of type 2 diabetes established by using a soft-pellet diet

**DOI:** 10.1038/s41598-024-57727-2

**Published:** 2024-04-01

**Authors:** Sayaka Akieda-Asai, Hao Ma, Wanxin Han, Junko Nagata, Fumitake Yamaguchi, Yukari Date

**Affiliations:** 1 Frontier Science Research Center, University of Miyazaki, Miyazaki, 889-1692 Japan; 2 Department of Sensory and Motor Organs, Faculty of Medicine, University of Miyazaki, Miyazaki, 889-1692 Japan; 3 Department of Nursing, Faculty of Medicine, University of Miyazaki, Miyazaki, 889-1692 Japan

**Keywords:** Muscle atrophy, Diabetes, Glucose metabolism, Energy metabolism, Soft food, Eating habits, Metabolic disorders, Experimental models of disease

## Abstract

Dietary factors such as food texture affect feeding behavior and energy metabolism, potentially causing obesity and type 2 diabetes. We previously found that rats fed soft pellets (SPs) were neither hyperphagic nor overweight but demonstrated glucose intolerance, insulin resistance, and hyperplasia of pancreatic β-cells. In the present study, we investigated the mechanism of muscle atrophy in rats that had been fed SPs on a 3-h time-restricted feeding schedule for 24 weeks. As expected, the SP rats were normal weight; however, they developed insulin resistance, glucose intolerance, and fat accumulation. In addition, skeletal muscles of SP rats were histologically atrophic and demonstrated disrupted insulin signaling. Furthermore, we learned that the muscle atrophy of the SP rats developed via the IL-6–STAT3–SOCS3 and ubiquitin–proteasome pathways. Our data show that the dietary habit of consuming soft foods can lead to not only glucose intolerance or insulin resistance but also muscle atrophy.

## Introduction

Obesity-associated insulin resistance is a major risk factor for type 2 diabetes^1,2^. However, in East Asia, the prevalence of type 2 diabetes is high, even with normal body mass index (BMI)^3,4^. In addition, a BMI of 23 or higher is a risk factor for insulin resistance and diabetes in Japanese patients^5^. One potential reason for this pattern is that East Asians are genetically predisposed to decreased insulin sensitivity and insulin response^6^. Furthermore, compared with Westerners, East Asians have a greater amount of body fat and a greater tendency to visceral adiposity at any given BMI^4^. In addition to high visceral adiposity, Asian patients with type 2 diabetes have increased accumulation of ectopic fat (e.g., in liver, skeletal muscle)^7,8^.

Recently, we investigated the effect of food texture on energy metabolism in rats by using standard pelleted chow (control pellets [CPs]; 51% carbohydrate, 25% protein, and 4.6% fat) or the same pellets to which we added water (soft pellets [SPs]). Despite the similarities in caloric intake and body weight between the two groups, the rats fed SPs on a 3-h time-restricted feeding schedule for 14 weeks showed glucose intolerance, insulin resistance with disruption of hepatic insulin signaling, and hyperplasia of pancreatic β-cells^8^. These findings suggest that the phenotype of the SP group may be similar to that of East Asian type 2 diabetes.

As a metabolic organ, skeletal muscle is one of the main tissues responsible for whole-body glucose homeostasis and lipid utilization. Hyperglycemia and insulin resistance—the central disorders of type 2 diabetes—contribute to the pathogenesis of skeletal muscle atrophy and dysfunction^9–12^. In fact, some reports indicate that diabetic patients lose approximately 26% more muscle mass and 33% more muscle strength annually than do their nondiabetic peers^13–16^. In the current study, first we histologically evaluated whether providing rats an SP diet on a 3-h time-restricted feeding schedule for 24 weeks led to atrophy of the gastrocnemius and soleus skeletal muscle. Next, we examined insulin signaling, glucose transporter 4 (GLUT4) translocation, and the expression of proinflammatory cytokines in the skeletal muscle of the SP group. Finally, we investigated various protein degradation pathways and their upstream factors in the skeletal muscle of SP rats.

## Materials and methods

### Ethics statement

All procedures were performed in accordance with the guidelines and regulations of University of Miyazaki. This study was also performed in accordance with the NIH guidelines (ARRIVE) for animal care and animal experimentation. The study protocol was approved by the Ethics Review Committee for Animal Experimentation of the Faculty of Medicine, University of Miyazaki (approval no. 2019-516).

### Animals and experimental design

The study involved 32 male Wistar rats (age, 7 weeks; Charles River Japan, Shiga, Japan). Rats were individually housed in plastic cages at a constant room temperature under a 12:12-h light:dark cycle (lights on, 08:00–20:00). The rats were randomly allocated to two groups (*n* = 16 per group) and fed either CPs (standard laboratory chow; 51% carbohydrate, 25% protein, and 4.6% fat; CE-2; CLEA Japan, Tokyo, Japan) or SPs (standard laboratory chow [1 kg] soaked in water [1.4 L]) for 24 weeks. The hardness of CPs exceeded 200 N, whereas that of SPs was 0.32 N. Prepared SPs were stored at 4 °C and used within 1 week; visible bacteria counts were similar between CPs and SPs, and no mold or yeast growth occurred. The hardness and bacteria tests were performed by Japan Food Research Laboratories (Tokyo, Japan). All rats were fed between 10:00 and 13:00 on a 3-h time-restricted feeding schedule. All animals had ad libitum free access to water throughout the study period, and body weight and caloric intake were monitored once each week. Rats were deeply anesthetized with pentobarbital sodium (100 mg/kg body weight, intraperitoneally) and euthanized by decapitation before blood and tissues were collected as a terminal procedure.

### Assessment of insulin resistance

Insulin resistance (HOMA-IR) in rats was determined by using obtained measurements of fasting insulin and blood glucose levels according to the formula^17^

$${\text{HOMA-IR}} = 26 \times {\text{fasting}}\;{\text{insulin}}\;({\text{ng/mL}}) \times {\text{fasting}}\;{\text{glucose}}\;({\text{mg/dL}}){/}405.$$

### Glucose tolerance test

Glucose tolerance testing was performed on the rats fed CPs or SPs for 16 weeks (*n* = 10 per group). Rats were fasted overnight; at 9:00 the following morning, each was injected intraperitoneally with glucose (2 g/kg body weight). To collect blood samples for measurement of glucose and insulin levels, xylocaine 2% jelly (lidocaine HCl; AstraZeneca, Osaka, Japan) was applied to the tail skin for surface anesthesia, after which blood was collected from a lanced tail vein into heparinized capillary tubes. Blood glucose was measured by using a handheld glucometer (Breeze 2, Bayer, Osaka Japan). The remaining samples were immediately centrifuged at 1500×*g* for 15 min at 4 °C, and plasma was stored at − 30 °C until insulin assay. Plasma insulin levels were measured by using an ELISA kit (Morinaga, Yokohama, Japan). The area under the curve (AUC) was calculated by using Prism 8 (GraphPad Software, San Diego, CA, USA).

### Plasma triglyceride level and triacylglycerol content

To measure triglyceride concentrations during feeding, we prepared plasma from blood that was collected from a lanced tail vein into heparinized capillary tubes, as described earlier (*n* = 5 per group). To assay hepatic and muscle triacylglycerol contents, 25 mg liver or 50 mg skeletal muscle was homogenized by using BioMasher (Nippi, Tokyo, Japan), and then lipids were extracted by using 500 ml ice-cold chloroform:methanol (2:1 [v/v]) as described previously^18^. The triacylglycerol contents of plasma, liver, and skeletal muscle samples were quantified by using a LabAssay Triglyceride kit (Wako Chemicals, Tokyo, Japan).

### Measurement of adiposity by using computed tomography (CT) analysis

Rats fed CPs or SPs for 24 weeks were anesthetized (pentobarbital, 50 mg/kg body weight intraperitoneally) and then scanned by micro-CT (LaTheta, Aloka, Tokyo, Japan) at 1.5-mm intervals from the proximal end of L1 to the distal end of L6 (*n* = 10 per group). Abdominal adiposity and lean mass were then determined by using LaTheta software (version 1.00).

### Cross-sectional area (CSA) of skeletal muscle fiber

The gastrocnemius and soleus muscle tissues from rats fed CPs or SPs for 24 weeks were cut at mid-belly, frozen in liquid nitrogen, and stored at − 80 °C. Serial cross-sections (thickness, 10 μm) were cut in a cryostat microtome. To measure the CSA of individual fibers, muscle cryostat sections were stained for laminin, a major component of the basal lamina. To this end, sections were fixed in 10% neutral buffered paraformaldehyde, permeabilized and blocked in PBS containing 0.3% Triton X and 5% (v/v) normal goat serum (Thermo Fisher Scientific, Waltham, MA, USA) for 30 min at room temperature. And then, the sections were incubated overnight at 4 °C with a primary antibody to laminin (dilution, 1:400; catalog no. L9393, Sigma-Aldrich, St. Louis, MO, USA), and then incubated with Alexa Fluor 488–conjugated goat anti-rabbit IgG (1:200; Thermo Fisher Scientific) for 40 min at room temperature; stained sections were mounted with ProLong Gold (Thermo Fisher Scientific). Stained muscle samples of CP and SP groups were imaged under a fluorescence microscope (All-in-One, Keyence, Osaka, Japan) and analyzed by using the manufacturer-supplied software (Keyensce).

### Western blotting

The gastrocnemius and soleus skeletal muscles were collected from rats fed CPs or SPs for 24 weeks (*n* = 6 per group) at 30 min after intraperitoneal injection of saline or insulin (1 U/kg body weight; Eli Lilly Japan, Kobe, Japan) or after overnight fasting. Muscle samples (25 mg each) were homogenized in mammalian cell lysis reagent (ProteoJET, Fermentas Life Sciences, Ontario, Canada) containing proteinase inhibitor cocktail (Roche Diagnostics, Basel, Switzerland) and phosphatase inhibitor cocktail (Roche Diagnostics). For GLUT4 analysis, subcellular fractionation was performed, and the plasma membrane (PM) fraction was collected (Mem-PER Plus membrane protein extraction kit, Thermo Fisher Scientific). Protein concentrations were determined by using a BCA Protein Assay Kit (Pierce, Rockland, IL, USA).

Each lane of an 8% (for Stat3), 10% (for Akt and GLUT4), or 15% (for IL-6 and β-actin) Tris–glycine SDS-PAGE gel contained 30 µg total protein. Proteins were transferred onto polyvinylidene difluoride membrane (Immobilon-P, Merck Millipore, Tokyo, Japan), which was blocked with 3% non-fat dry milk in 10 mM Tris–HCl (pH 7.5)/150 mM NaCl with 0.05% Tween 20 (TBST) for 1 h at room temperature. The membrane was incubated overnight at 4 °C in TBST containing 0.1% nonfat dry milk (w/v) and the following primary antibodies at a dilution of 1:2000: rabbit anti-Akt (Cell Signaling Technology, Danvers, MA, USA); rabbit anti-phospho-Akt (Ser473) (Cell Signaling Technology); rabbit anti-Stat3 (Cell Signaling Technology); rabbit anti-phospho-Stat3 (Thr705) (Cell Signaling Technology); rabbit anti-Glut4 (Cell Signaling Technology); rabbit anti-IL-6 (Sigma-Aldrich); and mouse anti-β-actin (MBL Life Sciences, Tokyo, Japan). Horseradish peroxidase–conjugated goat anti-rabbit IgG (H + L) (1:20,000; Epitomics, Burlingame, CA, USA) was applied as a secondary antibody. Chemiluminescence was quantified by using Western BLoT Quant HRP substrate (Takara Bio, Shiga, Japan) followed by image analysis (ImageQuant LAS-4000, GE Healthcare, Piscataway, NJ, USA). The amount of phospho-Akt, phospho-Stat3, IL-6, and GLUT4 in the PM fraction was quantified relative to total Akt, Stat3, β-actin, and GLUT4, respectively, by using densitometry (Image J, National Institutes of Health, Bethesda, MD, USA).

### Plasma IL-6 level

The IL-6 level in the plasma samples (prepared as described earlier) was quantified by using a rat IL-6 ELISA kit (Sigma-Aldrich).

### Cell culture

We obtained the L6 rat myoblast cell line from RIKEN Cell Bank (Tsukuba, Japan) and maintained the cells in growth medium (Dulbecco’s modified Eagle’s medium [DMEM]) containing 10% fetal bovine serum, GlutaMAX (Thermo Fisher Scientific), and 1% antibiotic–antimycotic solution (Nakarai Tesque, Kyoto, Japan) at 37 °C under 5% CO_2_ in air. To maintain their undifferentiated state, L6 cells were passaged before they achieved 80% confluence. The differentiation of L6 cells was induced by replacing the growth medium with differentiation medium (DMEM containing 5% horse serum [Thermo Fisher Scientific] and 1% antibiotic–antimycotic solution) at 100% confluence. To ensure they were fully differentiated, cells were exposed to differentiation medium for 4 days before they were used in experiments.

To obtain satellite cells, the extensor digitorum longus muscles were isolated from male rats (8 weeks old) and digested by using DMEM containing 0.2% type I collagenase (Wako Chemicals), GlutaMAX, and 1% antibiotic–antimycotic solution. Collected myofibers were confirmed under a high-power stereomicroscopy to be free of contaminating cells such as fibroblasts. Satellite cells were obtained from the isolated myofibers and cultured in mitogen-rich medium (DMEM containing 30% fetal bovine serum, GlutaMAX, 1% chick embryo extract ([USBiological, Salem, MA, USA], 10 ng/ml recombinant human basic fibroblast growth factor [154 amino acids, Nacalai Tesque, Kyoto, Japan], and 1% antibiotic–antimycotic solution) on iMatrix-511-coated dishes (Matrixome, Osaka, Japan). The cells were confirmed to express Pax7^19,20^. Cells were incubated at 37 °C under 5% CO_2_ in air, and media were changed every two 2 days.

L6 and satellite cells were preincubated in growth medium containing 5 mM glucose (basal medium) for 18 h and then incubated for 24 h in growth medium containing 10 mM glucose, 25 mM glucose, or 200 μM fatty acid (palmitic acid, Wako Chemicals). After this treatment, the cells were harvested in Sepasol-RNA I Super G (Nacalai Tesque) and stored at − 80 °C until RNA extraction.

### Quantitative PCR analysis

Gastrocnemius and soleus tissue was removed from CP or SP rats after 24 weeks (*n* = 10 per group). Total RNA was extracted by using Sepasol-RNA I Super G solution (Nacalai Tesque). First-strand cDNA was synthesized from total RNA by using a commercially available kit (PrimeScript RT reagent kit; Takara Bio). The resulting samples underwent quantitative PCR analysis for *atrogin1*, *Bnip3*, *casitas B lineage lymphoma protooncogene b* (*Cblb*), *cathepsin L* (*Ctsl*), *CD11c*, *CD68*, *interleukin* (*IL*)-*1β*, *IL-6*, *LC3*, *muscle-specific RING-finger protein 1* (*MuRF1*), *suppressor of cytokine signaling 3* (*SOCS3*) and *tumor necrosis factor* (*TNF*)-*α*. Quantitative PCR assays were conducted on a StepOnePlus Real-Time PCR System (Applied Biosystems, Waltham, CA, USA) by using SYBR Premix Ex Taq II reagents (Takara Bio). The relative abundances of all reaction products were normalized to the level of *hypoxanthine phosphoribosyltransferase I* (*HPRT*) mRNA, which was unaffected by experimental factors, and calculated by using the 2-delta delta CT method^21^.

In addition, total RNA was extracted from L6 and isolated satellite cells, transcribed into cDNA, and analyzed by quantitative PCR as described for skeletal muscle. The relative abundance of each reaction product was normalized against the level of *β-glucuronidase* (*Gusb*) mRNA (for L6 cells) or *tyrosine 3-monooxygenase/tryptophan 5-monooxygenase activation protein* (*Ywhaz*) mRNA (for satellite cells). The primer sets for all of these genes are listed in Table 1.

**Table 1:** Primers used for quantitative PCR analysis.

Gene	Accession no	Product size (bp)	Forward (5′ → 3′)	Reverse (5′ → 3′)
Atrogin1	NM_133521	138	TTGTGCGATGTTACCCAAGAAGA	GTGAAAGTGAGACGGAGCAGC
Bnip	NM_053420	69	CAGAGCGGGGAGGAGAACCTGCAG	GCTGCTCCCATTCCCATTGCTGAAC
Cblb	NM_133601	85	GCCCAACGCCCATATCTATTTAC	TTGATCACGCACTCGGTCAG
CD11c	NM_001419757	76	AAGCCCAAGTGTTCCTTCG	CACATGAGGTGCAGGGAGT
Ctsl	NM_013156	138	TATCGCCACCAGAAGCACAAGA	AGCCCAGCAAGAACCACACTG
CD68	NM_001031638	105	AGCAATTCACCTGGACCTGCT	AAGAGAGATTGGTCACTGGCG
IL-1β	NM_031512	235	TGTGATGAAAGACGGCACAC	GGGTTCCATGGTGAAGTCAAC
IL-6	NM_012589	114	CACTTCACAAGTCGGAGGCTT	TCTGACAGTGCATCATCGCTG
LC3	NM_022867	67	CACCCGCTGCTGTGTAGA	CCTGGACAGGGTTGGAACT
MuRF1	NM_080903	131	ATGCTGGTGGCAGGGAACGA	ATGGCGTAGAGGGCGTCAAA
SOCS3	NM_053565	65	AATCCAGCCCCAATGGTC	GGCCTGAGGAAGAAGCCTAT
TNF-α	NM_012675	141	ATCGGTCCCAACAAGGAGGA	CTCCGCTTGGTGGTTTGCTA
Gusb	NM_017015	60	CTGATCGACTGGCTGGATGGC	CCCCATGTCTGCGTCATATCT
HPRT	NM_012583	81	TCATGAAGGAGATGGGAGGC	CCAGCAGGTCAGCAAAGAACT
Ywhaz	NM_013011	81	TGGACATCGGATACCCAAGG	GGCAGACAAAGGTTGGAAGG

### Statistical analysis

We compared groups of data by using Student *t*-tests, Mann–Whitney U tests, and one-way or two-way analysis of variance with post hoc Tukey–Kramer tests (Prism 8, GraphPad Software). *P* values less than 0.05 were considered to be significant. Data are reported as means ± SEM.

## Results

### Body weight and caloric intake

**Figure 1:**
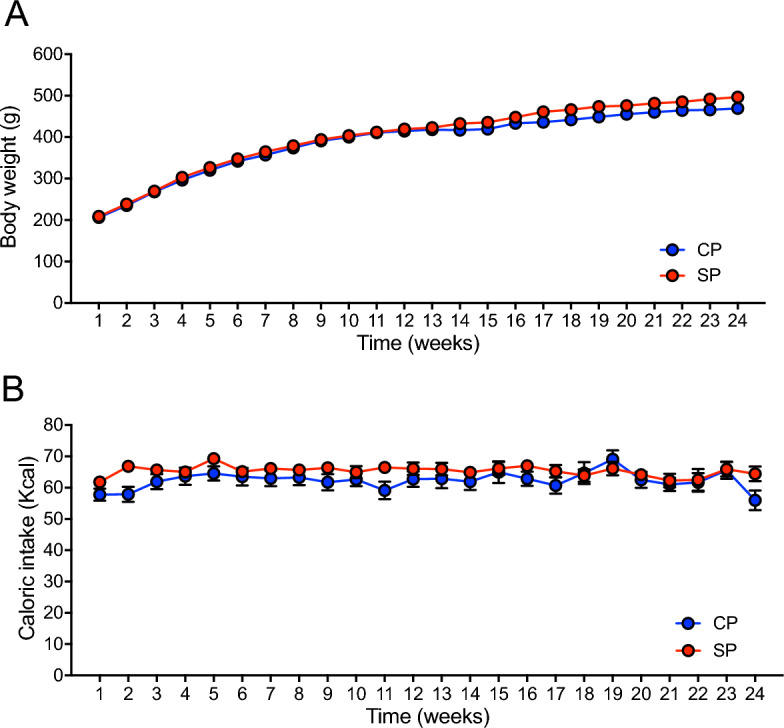
Body weight and caloric intake. (**A**) Body weight and (**B**) caloric intake of rats fed soft pellets (SPs) or control pellets (CPs) (*n* = 16 per group).

We previously showed that neither body weight nor caloric intake differed between CP and SP rats fed the respective diets for 14 weeks^8^. We investigated the body weight and caloric intake of rats fed SPs or CPs for 24 weeks by measuring these parameters monitored body weight and caloric intake every week. Body weight (Fig. 1A) and caloric intake (Fig. 1B) remained similar between the CP and SP groups throughout the 24-week feeding period.

### Effect of SP diet on glucose tolerance

**Figure 2:**
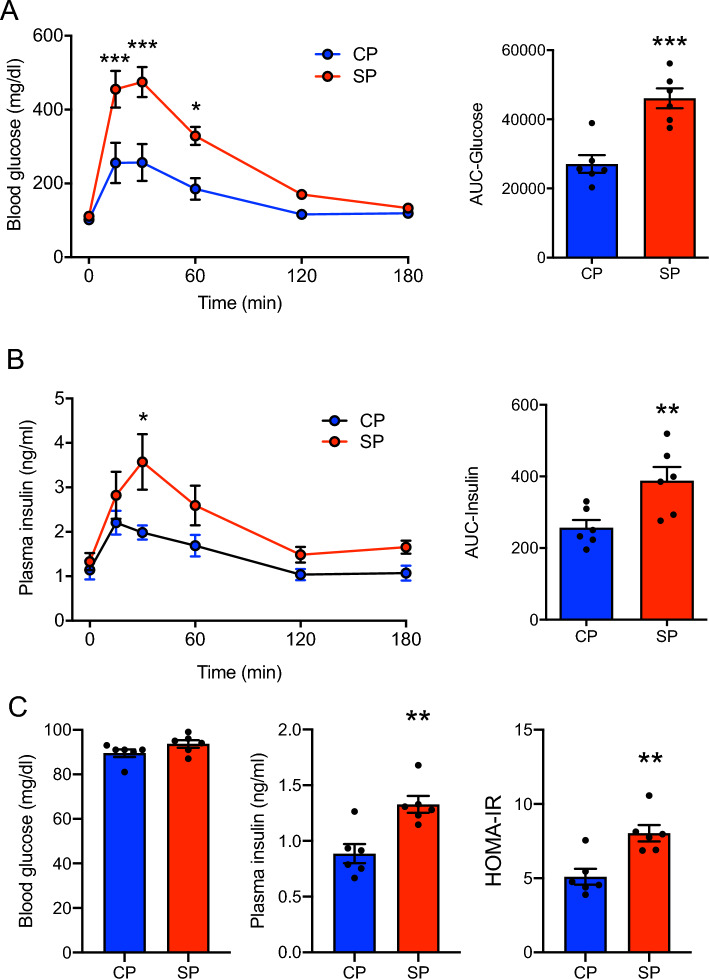
Glucose, insulin, and HOMA-IR. (**A**) Blood glucose levels assessed through glucose tolerance testing (GTT) of rats fed SPs or CPs for 16 weeks. (**B**) Plasma insulin levels assessed through GTT. (**C**) Blood glucose, plasma insulin level, and HOMA-IR were assessed in overnight-fasted rats fed SPs or CPs for 24 weeks. *n* = 6 per group. *P < 0.05, **P < 0.01, ***P < 0.01 vs. rats fed CPs. AUC, area under the curve.

Glucose tolerance testing showed that blood glucose levels in the SP group were significantly (*P* < 0.05) higher at 15, 30, and 60 min after glucose injection than those in the CP group (Fig. 2A), and the insulin levels in the SP group were significantly higher at 30 min (Fig. 2B). The AUCs for the glucose and insulin responses were greater for the SP group than the CP group (Fig. 2A,B). In addition, the overnight fasting blood glucose level did not differ between the CP and SP groups, whereas plasma insulin level and HOMA-IR were significantly higher in the SP group (Fig. 2C).

### Subcutaneous and abdominal fat accumulation, hepatic and muscle triacylglycerol content, and plasma triglyceride concentration

**Figure 3:**
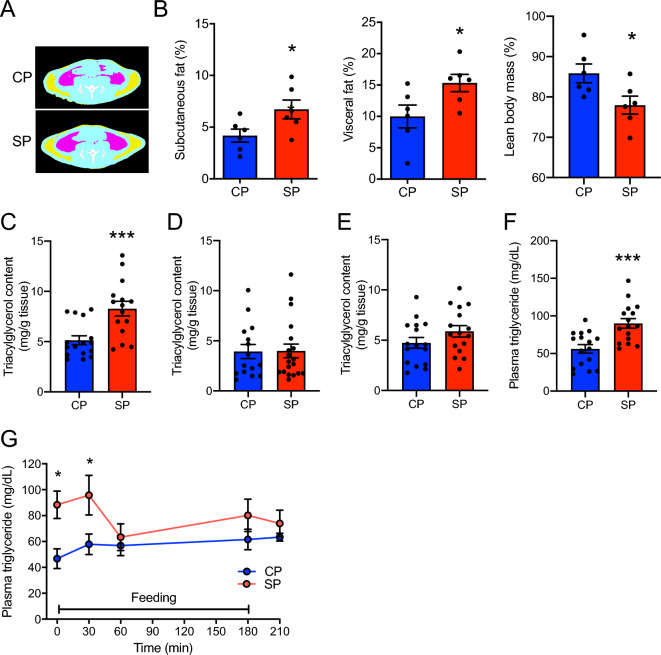
Subcutaneous and visceral fat proportions and lipid content in liver and blood of rats fed SPs or CPs for 24 weeks. (**A**) Computed tomographic images of rats fed SPs or CPs, indicating areas of subcutaneous (yellow) and visceral (red) fat and muscle (blue). (**B**) Percentages of subcutaneous fat, visceral fat, and lean mass as estimated from images taken from the proximal end of L1 to the distal end of L6. *n* = 6 per group. (**C**–**E**) Triacylglycerol content in (**C**) liver, (**D**) gastrocnemius muscle, and (**E**) soleus muscle. (**F**) Plasma triglyceride concentration. *n* = 16 per group. (**G**) Plasma triglyceride levels after feeding. *n* = 5 per group. *P < 0.05, ***P < 0.001 vs. rats fed CPs.

Subcutaneous or visceral adipose tissue was identified automatically on CT slices (Fig. 3A). Although body weight and caloric intake did not differ between the two groups, the percentages of both subcutaneous and visceral fat were significantly greater in the SP group than in the CP group (Fig. 3B). In contrast, the lean mass was lower in the SP group than in CP group (Fig. 3B). In addition, the SP group had more hepatic triacylglycerol and higher plasma triglyceride concentrations than did the CP group (Fig. 3C,F), whereas the triacylglycerol contents in both the gastrocnemius and soleus muscles did not differ between the CP and SP groups (Fig. 3D,E). Plasma triglyceride levels at 0 and 30 min after feeding were significantly higher in the SP group than in the CP group (Fig. 3G).

### Insulin signaling and GLUT4 translocation to the PM in skeletal muscle after insulin injection

**Figure 4:**
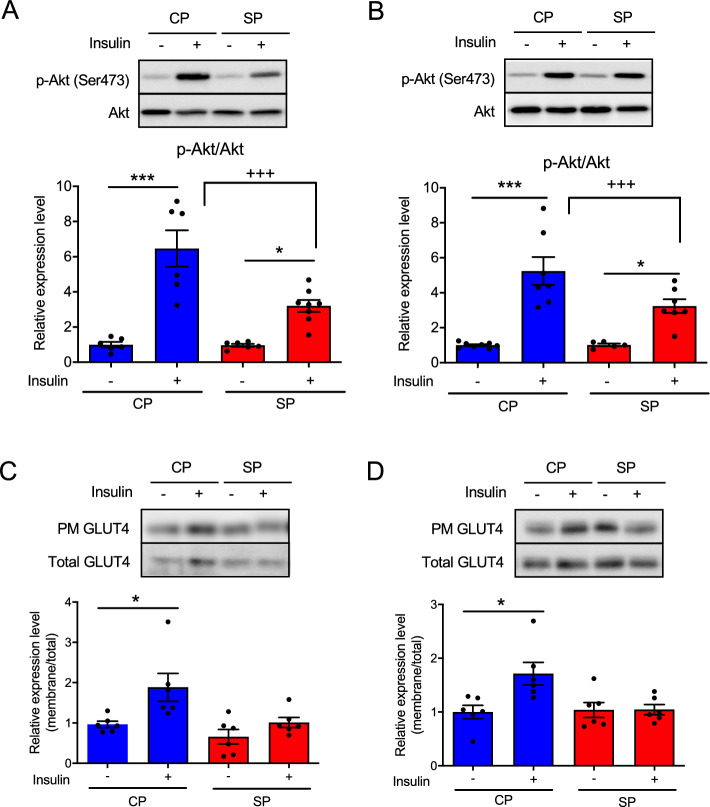
Amounts of phosphorylated (p-) Akt and GLUT4 in the skeletal muscle of rats fed SPs or CPs for 24 weeks. Quantities of p-Akt after insulin injection or without insulin treatment in (**A**) gastrocnemius muscle and (**B**) soleus muscle. The GLUT4 level in the plasma membrane (PM) fractions from (**C**) gastrocnemius muscle and (**D**) soleus muscle was assessed after insulin injection or without insulin treatment. The original gel blots are presented in the Supplementary Fig. S4. *n* = 6 per group. *P < 0.05, ***P < 0.001 vs. rats fed CPs or SPs without insulin treatment. ^+++^*P* < 0.001 vs. rats fed CPs and treated with insulin.

We investigated the amounts of phosphorylated Akt in skeletal muscle (gastrocnemius and soleus) and of phospho-GLUT4 in the PM after injecting insulin into rats fed SPs or CPs for 24 weeks. In both muscles, the amount of phosphorylated Akt was significantly greater after insulin injection than after saline injection in both groups of rats, but the level of phospho-Akt after insulin injection was significantly lower in SP group than in the CP group (Fig. 4A,B). In addition, GLUT4 translocation to the PM in both the gastrocnemius and soleus muscles after insulin stimulation was increased in the CP group but not in the SP group (Fig. 4C,D).

### Atrophy of and protein degradation pathways in skeletal muscle

**Figure 5:**
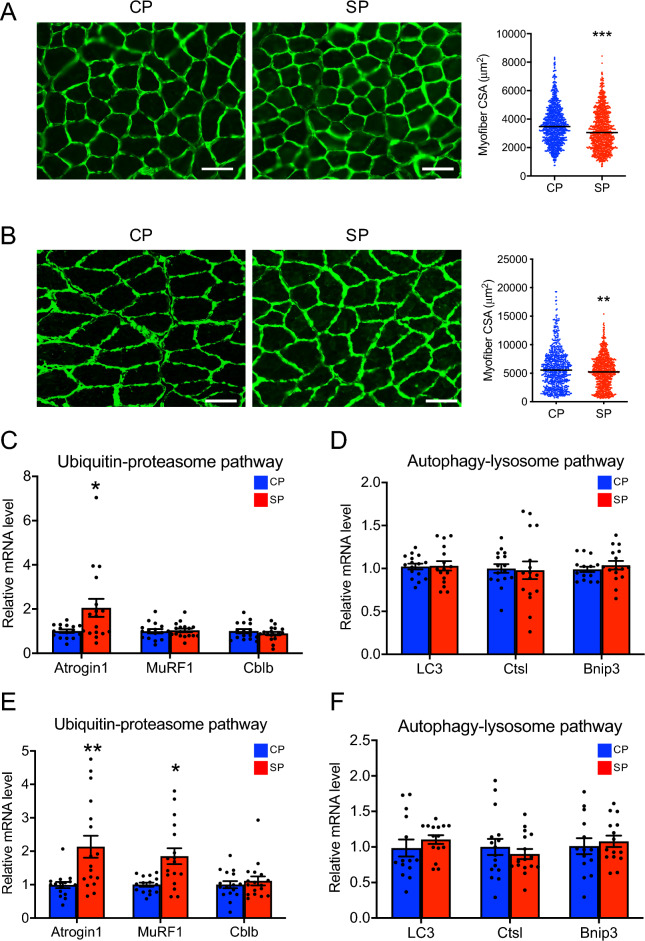
Cross-sectional area (CSA) of muscle fiber and assessment of protein degradation pathways in the skeletal muscle of rats fed SPs or CPs for 24 weeks. (**A**,**B**) Muscle fiber CSA in (**A**) gastrocnemius muscle and (**B**) soleus muscle. (**C**,**E**) mRNA levels of enzymes in the ubiquitin–proteasome pathway in (**C**) gastrocnemius muscle and (**E**) soleus muscle. (**D**,**F**) mRNA levels of enzymes in the autophagy–lysosome pathway in (**D**) gastrocnemius muscle and (**F**) soleus muscle. Bar, 100 μM. *n* = 16 per group. *P < 0.05, **P < 0.01, ***P < 0.001 vs. rats fed CPs.

Using histologic examination, we found that the myofiber CSA of the gastrocnemius and soleus muscles was significantly smaller in the SP group than the CP group (Fig. 5A,B). The ubiquitin–proteasome and autophagy–lysosome pathways are the two main pathways involved in the protein degradation of skeletal muscle atrophy^22–25^. We examined the expression levels of several enzymes in the ubiquitin–proteasome pathway—Atrogin1, MuRF1, and Cblb—and in the autophagy-lysosome pathway—LC3, Ctsl, and Bnip3—in the skeletal muscle of the CP and SP groups. The amount of *atrogin1* mRNA in the gastrocnemius muscle was significantly greater in the SP group than in the CP group, whereas mRNA levels of *MuRF1* and *Cblb* did not differ between groups (Fig. 5C). In soleus muscle, *atrogin1* and *MuRF1* mRNA levels were significantly higher in the SP group than in the CP group, but *Cblb* mRNA quantities were similar between groups (Fig. 5E). In both muscles, the mRNA levels of *LC3*, *Ctsl*, and *Bnip3* did not differ between the CP and SP groups (Fig. 5D,F).

### Trigger factor for protein degradation and its downstream pathway in skeletal muscle

**Figure 6:**
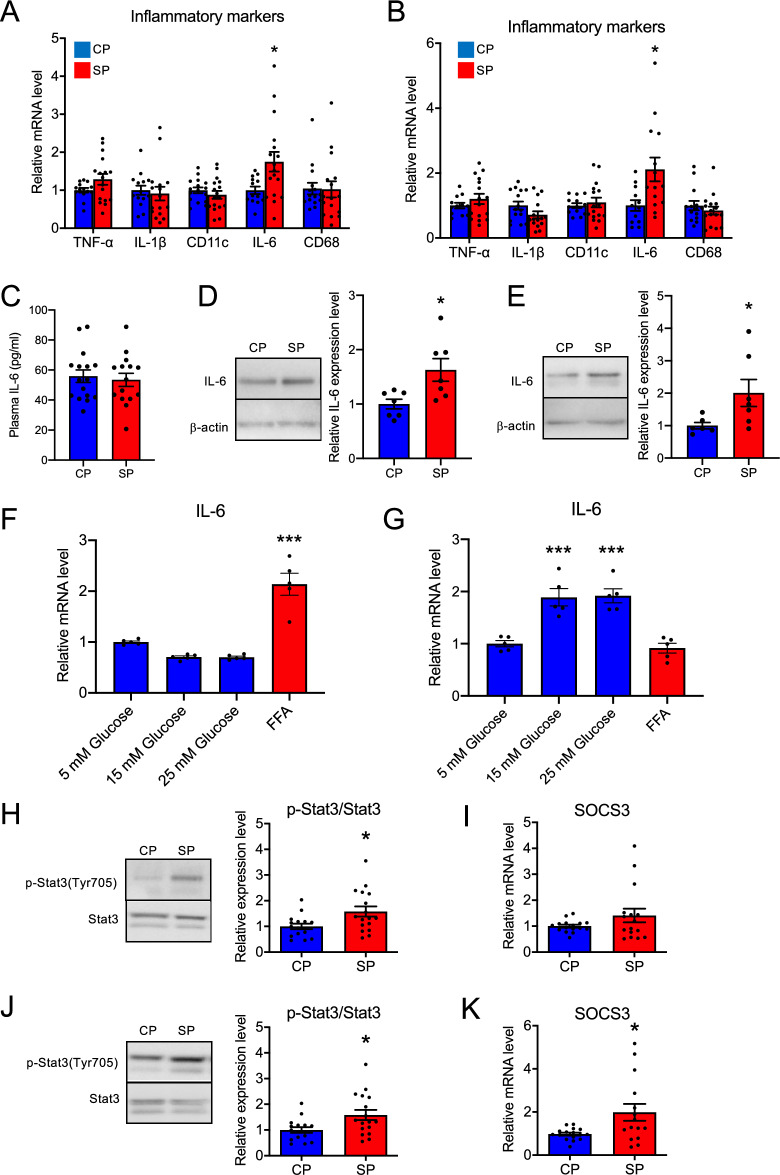
Proinflammatory factors and the IL-6–STAT3–SOCS3 pathway in the skeletal muscle of rats fed SPs or CPs for 24 weeks. (**A**,**B)** Proinflammatory factors in (**A**) gastrocnemius muscle and (**B**) soleus muscle. *n* = 16 per group. (**C**) Plasma IL-6 levels. *n* = 16 per group. (**D**,**E**) IL-6 protein levels in (**D**) gastrocnemius muscle and (**E**) soleus muscle. *n* = 10 per group. (**F**) *IL-6* mRNA levels in L6 myocytes after treatment with glucose or free fatty acids (FFA) for 24 h. (**G**) *IL-6* mRNA levels in isolated satellite cells after treatment with glucose or FFA for 24 h. *n* = 5 per group. (**H**,**J**) Amount of p-Stat3 in (**H**) gastrocnemius muscle and (**J**) soleus muscle. The original gel blots are presented in the Supplementary Fig. S5. (**I**,**K**) *SOCS3* mRNA levels in (I) gastrocnemius muscle and (K) soleus muscle. *n* = 16 per group. *P < 0.05 vs. rats fed CPs. ***P < 0.001 vs. cells treated with 5 mM glucose.

Various proinflammatory factors, including TNFα, IL-1, and IL-6, promote skeletal muscle atrophy and are upregulated in muscle obtained from patients with type 2 diabetes^26–28^. Therefore, we evaluated the expression levels of several proinflammatory factors in skeletal muscle collected from CP and SP rats. Although the mRNA expression of *TNFα*, *IL-1β*, *CD11c*, and *CD68* did not differ between the CP and SP groups, the *IL-6* mRNA level was significantly higher in the SP group than in the CP group in both the gastrocnemius muscle (Fig. 6A) and soleus muscle (Fig. 6B). Plasma IL-6 levels did not differ between groups (Fig. 6C), whereas IL-6 protein expression in skeletal muscle was greater in SP rats (Fig. 6D,E). Next we investigated whether glucose or free fatty acid (FFA) induced the expression of IL-6 in L6 myocytes and isolated satellite cells. Treatment with FFA significantly increased *IL-6* mRNA levels of L6 myocytes, but glucose did not (Fig. 6F). In contrast, in isolated satellite cells, treatment with 15 or 25 mM glucose—but not FFA—significantly increased *IL-6* mRNA levels (Fig. 6G).

STAT3 and SOCS3 play important roles in the downstream pathway of IL-6 and are associated with muscle atrophy in obesity and diabetes^29,30^. To investigate the downstream pathway of IL-6, we examined the phosphorylation of Stat3 and mRNA expression of *SOCS* in skeletal muscle from the CP and SP groups. In both the gastrocnemius muscle (Fig. 6H) and soleus muscle (Fig. 6J), the amount of phospho-Stat3 was significantly greater in the SP group than in the CP group. The amount of *SOCS3* mRNA in the gastrocnemius was similar between the two groups but was numerically higher in the SP group than in the CP group (Fig. 6I); the amount of *SOCS3* mRNA in the soleus muscle was significantly higher in the SP group than the CP group (Fig. 6K).

## Discussion

In the present study, we created a rat model of type 2 diabetes without overweight or hyperphagia to investigate the mechanisms underlying the muscle atrophy associated with diabetes. We previously found that rats fed SPs on a 3-h-restricted schedule for at least 14 weeks show insulin resistance and glucose intolerance^18^. Therefore, we performed glucose tolerance testing in rats fed SPs for 16 weeks and confirmed that the SP rats we used in the current study presented type 2 diabetes. Furthermore, we had obtained data that the levels of various enzymes and cytokines involved in muscle degradation or atrophy did not differ between CP rats and those fed SPs for 4 or 14 weeks (Supplementary Figs. S1, S2) but were altered in rats fed SPs for 24 weeks. Therefore, we consider that rats fed an SP diet on a 3-h time-restricted schedule for 24 weeks is an appropriate model of normal-weight type 2 diabetes with muscle atrophy. As shown here, body weight did not differ between the SP and CP (control) groups, whereas the SP group had markedly higher percentages of visceral and subcutaneous fat and significantly lower percentages of lean body. These data indicate that the body composition of the SP group differed substantially from that of CP group, despite their similar body weights and caloric intakes. In addition, as assessed with fasting blood glucose and insulin levels, the SP group had overt glucose intolerance and elevated HOMA-IR, a key indicator of insulin resistance. Furthermore, compared with their control counterparts, the SP group showed significantly increased triglycerides in plasma and triacylglycerol contents in visceral fat and liver. Because an increased plasma triglyceride level is a well-known feature of the dyslipidemia associated with type 2 diabetes^31^, we consider that the increased triacylglycerol content of the SP group is strongly related to type 2 diabetes. In summary, rats fed SPs for 24 weeks presented with type 2 diabetes characterized by normal weight, glucose intolerance, insulin resistance, fat accumulation, and dyslipidemia.

Insulin regulates glucose uptake into muscle by inducing the translocation of GLUT4 from intracellular storage sites to the PM^32^. Here we investigated the amount of phospho-Akt in skeletal muscle and the translocation of GLUT4 into skeletal muscle PM after insulin injection. Although it increased in response to insulin injection, the amount of phospho-Akt in the skeletal muscle of the SP group was significantly lower than that of the CP group. This finding suggests that insulin-driven signal transduction in skeletal muscle may be reduced in the SP group. In addition, insulin injection failed to induce GLUT4 translocation in skeletal muscle PM in the SP group. Translocation of GLUT4 is thought to be a response to both Akt-dependent and -independent signals^33,34^. In particular, AMPK modulates the translocation of GLUT4 to the PM after muscle contraction in an insulin-independent manner^35,36^. In the current study, we demonstrated that the myofiber CSA was significantly smaller in the SP group than in the CP group. This finding indicates that the SP rats, a model of type 2 diabetes without hyperphagia and overweight, can develop diabetic muscle atrophy, resulting in weakened muscle contraction. Therefore, our results suggest that the impaired translocation of GLUT4 to the skeletal muscle PM of the SP group may be due to decreased insulin-associated signal transduction and to muscle atrophy.

Two protein degradation pathways are considered to be important in muscle loss (atrophy): the autophagy–lysosomal pathway and the ubiquitin–proteasome pathway^22–25^. Here we investigated mechanisms that contributed to the muscle atrophy of the SP group. The mRNA expression of enzymes in the ubiquitin–proteasome pathway was higher in the SP group than in the CP group, but that of enzymes in the autophagy–lysosomal pathway did not differ between the groups. These results suggest that the muscle atrophy of the SP group is mediated by protein degradation through the ubiquitin–proteasome pathway. Furthermore, the overproduction of proinflammatory cytokines accelerates muscle atrophy via the ubiquitin–proteasome pathway^23,37^. Indeed, the mRNA level of the proinflammatory marker *IL-6* was higher in the skeletal muscle of the SP group than in that of the CP group. As mentioned earlier, rats fed SPs for 14 weeks showed both glucose intolerance and insulin resistance^18^, but the mRNA expression of enzymes in the ubiquitin–proteasome pathway and IL-6 mRNA expression in skeletal muscle did not differ between CP and SP groups (Supplementary Figs. S1, S2). These findings imply that the persistently increased blood glucose and triglyceride levels in SP rats affect IL-6 metabolism in skeletal muscle, leading to muscle atrophy.

In the present study, neither the mRNA level of the macrophage marker *CD68* nor the triacylglycerol content in skeletal muscle differed between the CP and SP groups. These results suggest that the elevated *IL-6* expression in the skeletal muscle of the SP group did not originate from macrophage infiltration that accompanied lipid accumulation. Furthermore, plasma IL-6 levels were similar between groups, suggesting that IL-6 is not hypersecreted into the circulation during SP-induced type 2 diabetes. In skeletal muscle, IL-6 is produced not only by infiltrating macrophages but also by myocytes and satellite cells^37,38^. When we examined the effects of glucose and fatty acid on *IL-6* mRNA induction in myocytes or satellite cells, we found that high fatty acid concentration upregulated *IL-6* mRNA expression in myocytes but high glucose treatment increased its expression in satellite cells. In addition, the protein level of IL-6 in skeletal muscle was higher in SP rats than in CP rats. Although IL-6 effects on the central nervous system can modulate locomotor activity^39,40^, locomotor activity did not differ between the CP and SP groups (Supplementary Fig. S3). Furthermore, the plasma triglyceride levels of SP rats were markedly increased at 0 and 30 min during feeding. These results suggest that IL-6 levels in skeletal muscle are upregulated due to elevated blood triglycerides or glucose intolerance or both and may be an important trigger for inducing muscle atrophy in SP rats.

Chronic and low-level elevations of IL-6 are known to induce skeletal muscle atrophy^26^. In addition, local infusion of IL-6 into the skeletal muscle of rats induces STAT3 phosphorylation and SOCS3 transcription, which correlate with a local reduction in myofibrillar protein content^26^. Similarly, the skeletal muscle of our SP group showed elevated levels of phospho-Stat3 and of enzymes in the ubiquitin–proteasome pathway and increased transcription of *SOCS3* and *IL-6*. Together, these results suggest that skeletal muscle protein in the SP rats is degraded through the ubiquitin–proteasome pathway via IL-6–STAT3–SOCS3 signaling. In this regard, KLF15, a transcription factor, has recently been shown to induce muscle atrophy in skeletal muscle^41,42^. Considering that IL-6 is a downstream effector of KLF15^41^, the increase in IL-6 in the SP group may be regulated by KLF15. The mechanism through which IL-6 is upregulated in the SP group warrants further investigation.

In summary, we demonstrated here that, compared with controls, rats fed an SP diet for 24 weeks on a 3-h time-restricted schedule had glucose intolerance, insulin resistance, and visceral and hepatic fat accumulation despite the absence of overweight and hyperphagia. Furthermore, we revealed that the muscle atrophy in the SP rats occurs via IL-6–STAT3–SOCS3 signaling and the ubiquitin–proteasome pathway. These combined results suggest that the dietary habit of consuming soft foods contributes to both the onset of type 2 diabetes and its associated muscle atrophy, which is due, at least in part, to an increase in IL-6 induced by elevated blood glucose or triglyceride, or both. We previously confirmed that SP rats have significantly less food remaining in their stomachs than do CP rats, and we consider that the nutrient load reaches the intestine faster in SP rats^18^. Furthermore, we consider that 3-h time-restricted feeding would be also a critical factor to accelerate the onset of type 2 diabetes, because we preliminary examined insulin resistance in rats fed SPs ad libitum and found that it takes at least 27 weeks for them to show insulin resistance. However, precisely how highly absorbable foods such as SPs cause type 2 diabetes remains unknown. To elucidate the link between the digestive system and glucose metabolism, we intend to investigate the molecular and functional mechanisms involved in SP diet-induced type 2.

### Supplementary Information

Supplementary Information.

## Data Availability

The datasets generated during and/or analyzed during the current study are available from the corresponding author on reasonable request.
