# Supplementary material for: Mechanism of muscle atrophy in a normal-weight rat model of type 2 diabetes established by using a soft-pellet diet
Source: Sci Rep. 2024 Apr 1;14:7670. doi: 10.1038/s41598-024-57727-2 (PMC10984920; doi:10.1038/s41598-024-57727-2)
Supplement: Supplementary file 1 — Supplementary Information. [file 41598_2024_57727_MOESM1_ESM.docx]

**Supplementary Information for**

**Mechanism of muscle atrophy in a normal-weight rat model of type 2 diabetes established by using a soft-pellet diet**

Sayaka Akieda-Asai^1,*^, Hao Ma^1^, Wanxin Han^1^, Junko Nagata^2^, Fumitake Yamaguchi^1,3^, Yukari Date^1,*^

Corresponding Author Email: dateyuka@med.miyazaki-u.ac.jp

**This file includes:**

Supplemental Methods

Supplementary Figures

**Supplemental Methods**

**Locomotor activity**

Anesthesia of rats fed CP or SP for 22 weeks was induced by using 3% isoflurane and maintained with 1.5% isoflurane. Telemetric transmitters (HD-S10, Data Science International, St. Paul, MN, USA) were embedded in the intraperitoneal space by suturing them to the muscular layer of the abdominal wall. The rats were allowed to recover for at least 1 week before 24-hour recording of locomotor activity was initiated.

**
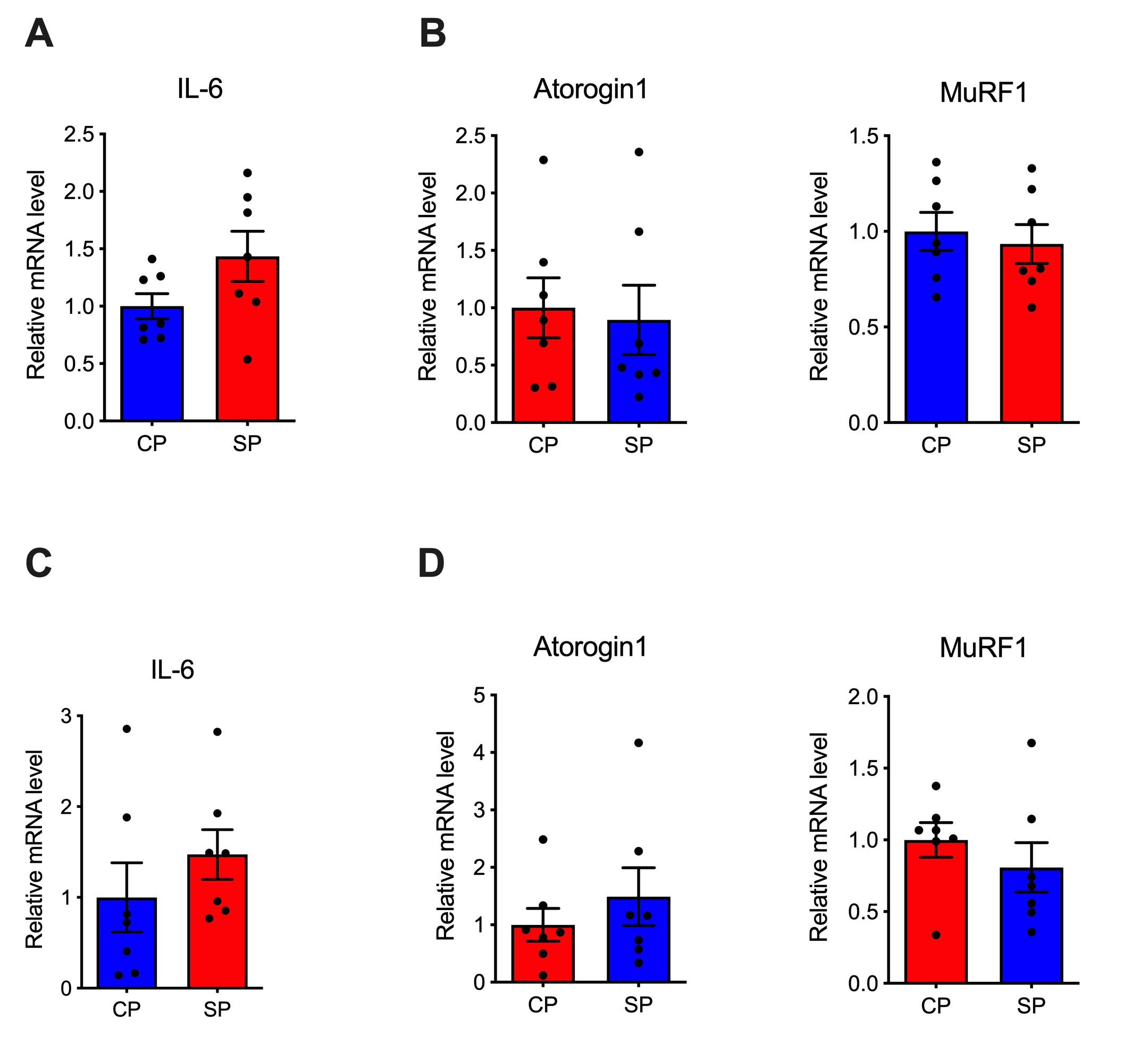
**

**Supplementary Figure S1:** IL-6 levels and assessment of protein degradation pathways in the skeletal muscle of rats fed SP or CP for 4 weeks. (**A, C)** *IL-6* mRNA levels in (A) gastrocnemius muscle and (C) soleus muscle. (**B, D**) mRNA levels of enzymes in the ubiquitin–proteasome pathway in (B) gastrocnemius muscle and (D) soleus muscle. *n* = 7 per group. Data are reported as means ± SEM.


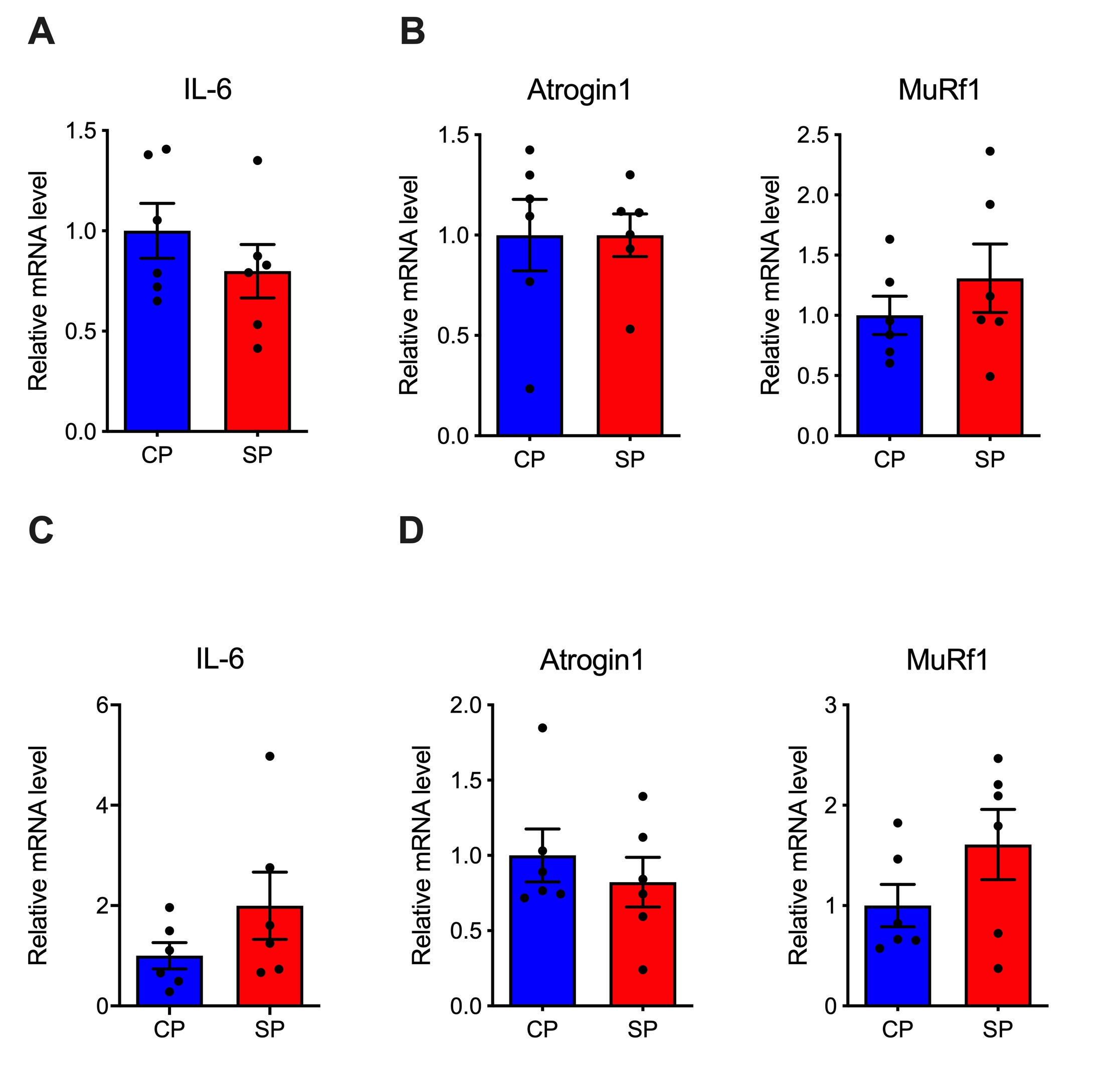


**Supplementary Figure S2:** IL-6 levels and assessment of protein degradation pathways in the skeletal muscle of rats fed SP or CP for 14 weeks. (**A, C)** *IL-6* mRNA levels in (A) gastrocnemius muscle and (C) soleus muscle. (**B, D**) mRNA levels of enzymes in the ubiquitin–proteasome pathway in (B) gastrocnemius and (D) soleus muscle. *n* = 6 per group. Data are reported as means ± SEM.


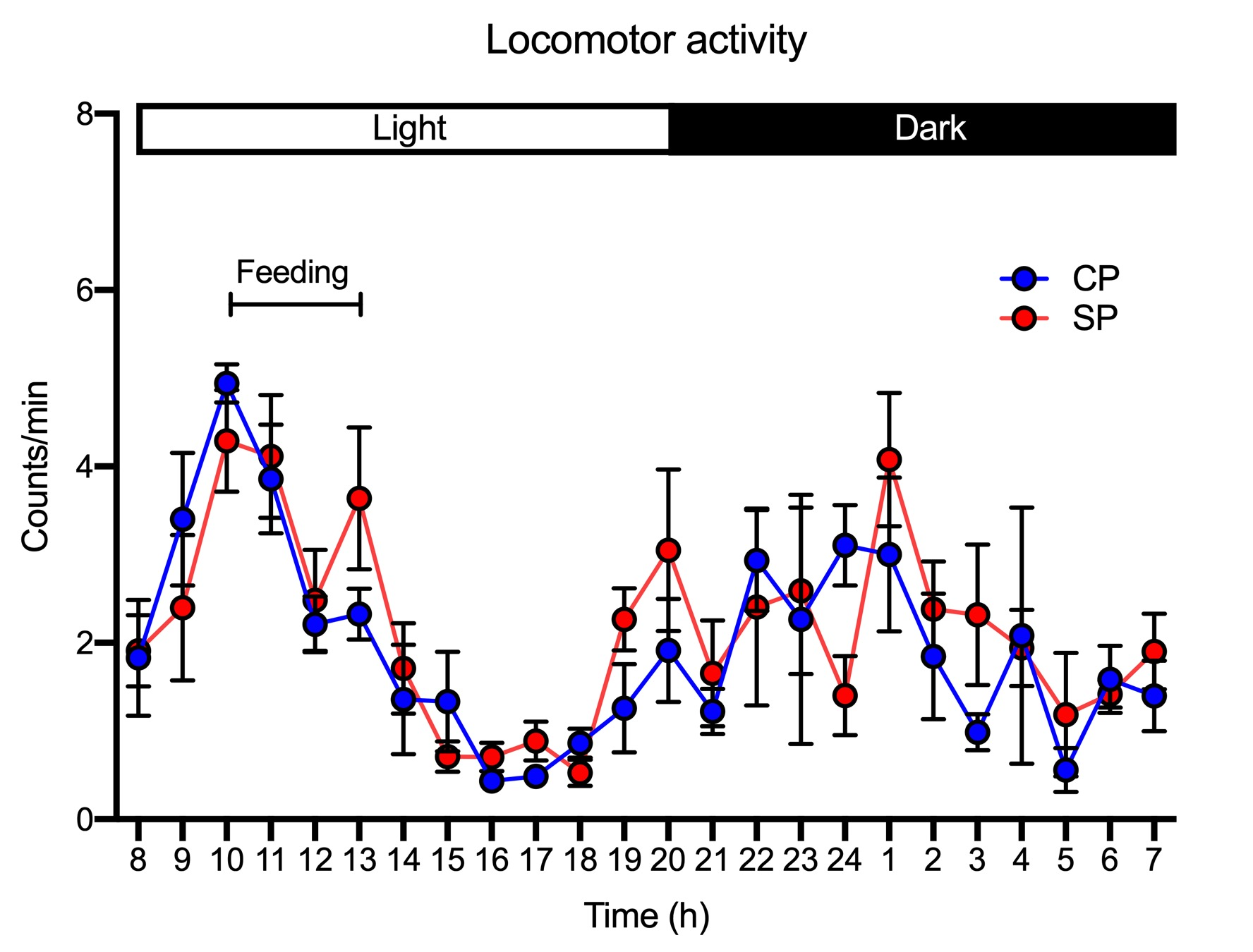


**Supplementary Figure S3:** Locomotor activity. *n* = 4 per group. Data are reported as means ± SEM.


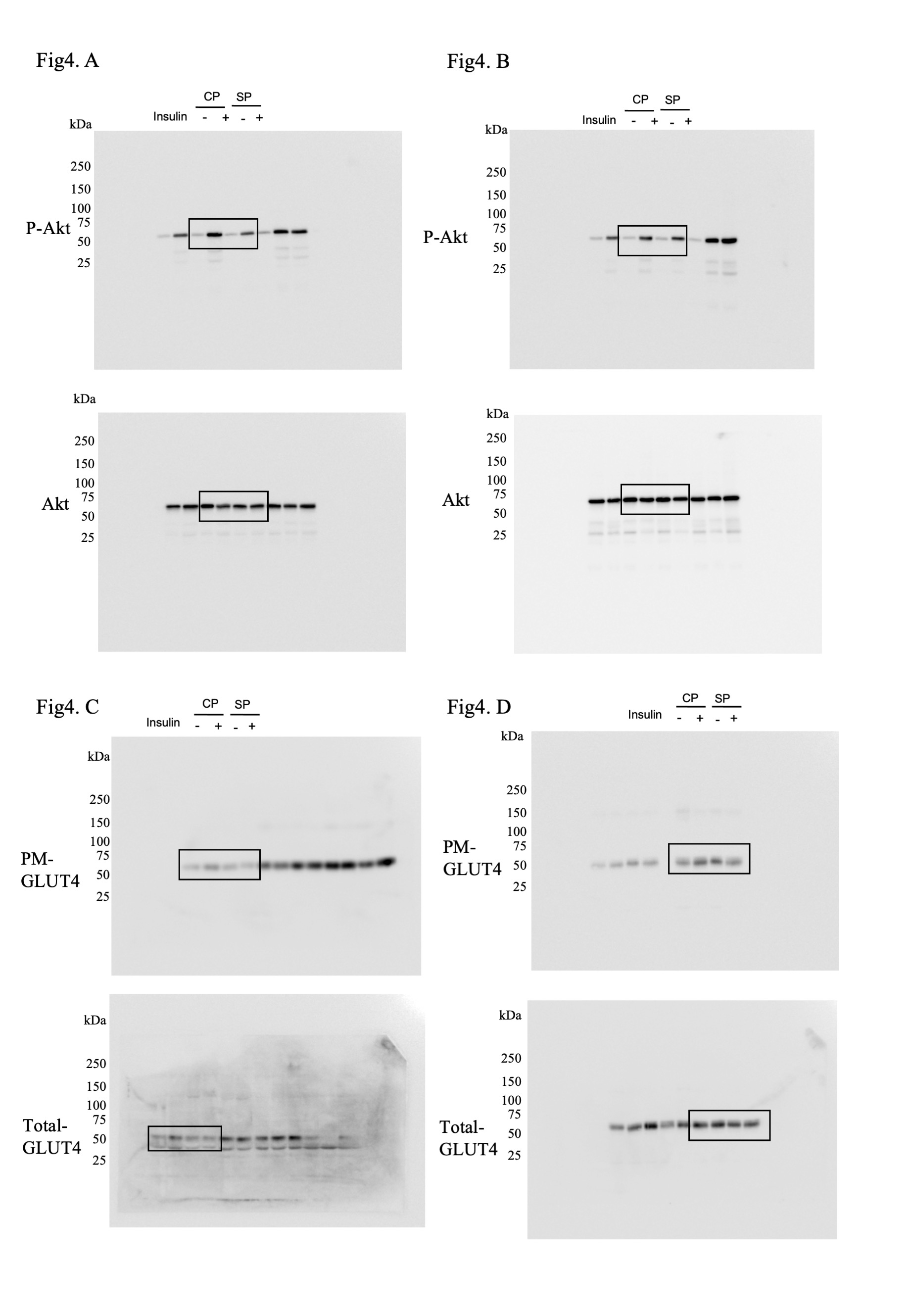


**Supplementary Figure S4:** Uncropped full-length pictures of western blotting membranes presented in the main Fig. 4A-4D.


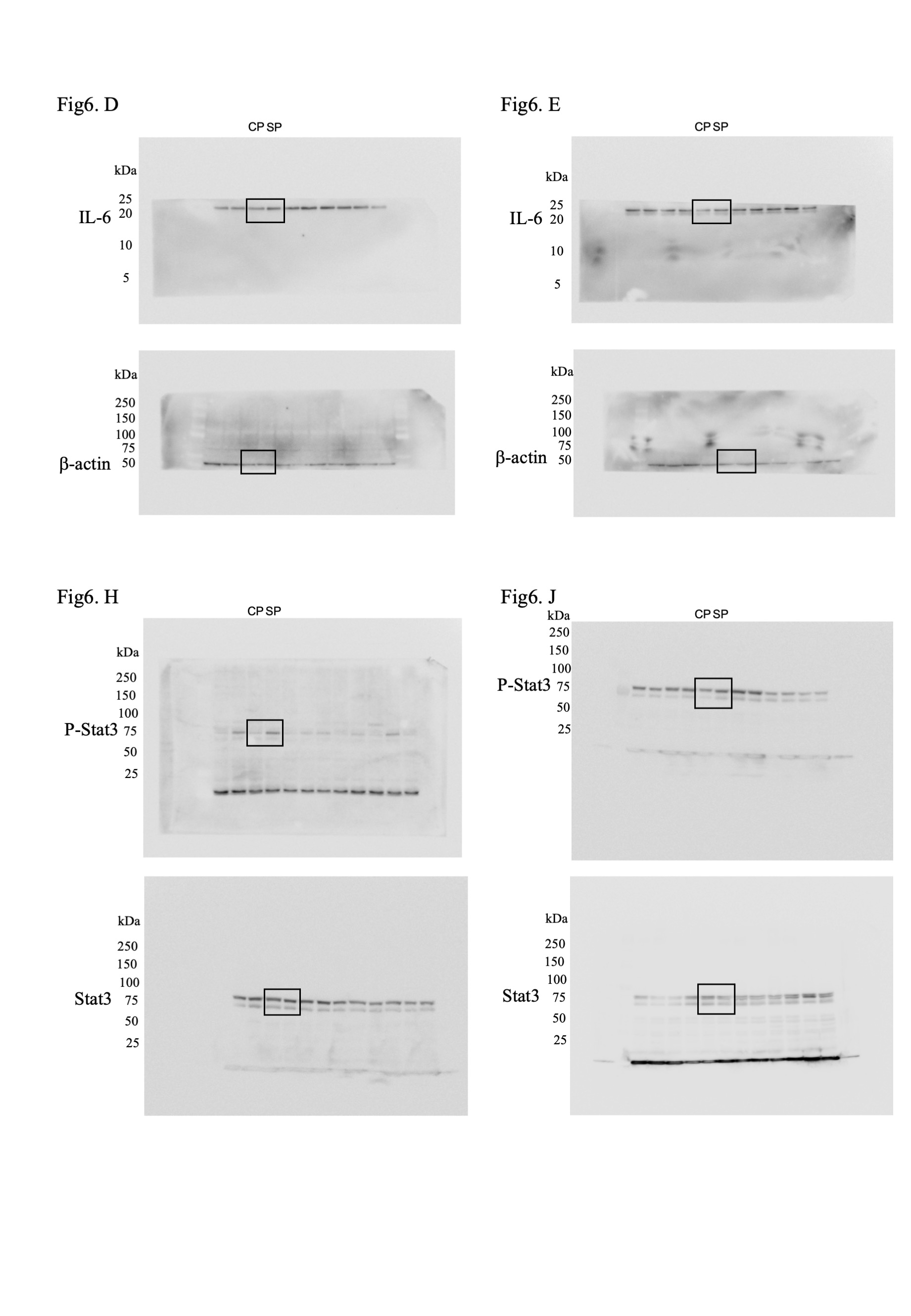
**Supplementary Figure S5:** Uncropped full-length pictures of western blotting membranes presented in the main Fig. 6D, 6H, 6IH and 6J. The blots of Fig. 6D and 6E were cut prior to hybridisation with antibodies.
